# Cerebral blood flow quantification using vessel-encoded arterial spin labeling

**DOI:** 10.1038/jcbfm.2013.129

**Published:** 2013-08-07

**Authors:** Thomas W Okell, Michael A Chappell, Michael E Kelly, Peter Jezzard

**Affiliations:** 1 Nuffield Department of Clinical Neurosciences, Centre for Functional Magnetic Resonance Imaging of the Brain, University of Oxford, Oxford, UK; 2 Department of Engineering, Institute of Biomedical Engineering, University of Oxford, Oxford, UK

**Keywords:** ASL, brain imaging, cerebral blood flow measurement, cerebral hemodynamics, MRI

## Abstract

Arterial spin labeling (ASL) techniques are gaining popularity for visualizing and quantifying cerebral blood flow (CBF) in a range of patient groups. However, most ASL methods lack vessel-selective information, which is important for the assessment of collateral flow and the arterial supply to lesions. In this study, we explored the use of vessel-encoded pseudocontinuous ASL (VEPCASL) with multiple postlabeling delays to obtain individual quantitative CBF and bolus arrival time maps for each of the four main brain-feeding arteries and compared the results against those obtained with conventional pseudocontinuous ASL (PCASL) using matched scan time. Simulations showed that PCASL systematically underestimated CBF by up to 37% in voxels supplied by two arteries, whereas VEPCASL maintained CBF accuracy since each vascular component is treated separately. Experimental results in healthy volunteers showed that there is no systematic bias in the CBF estimates produced by VEPCASL and that the signal-to-noise ratio of the two techniques is comparable. Although more complex acquisition and image processing is required and the potential for motion sensitivity is increased, VEPCASL provides comparable data to PCASL but with the added benefit of vessel-selective information. This could lead to more accurate CBF estimates in patients with a significant collateral flow.

## Introduction

Since its introduction,^1, 2^ arterial spin labeling (ASL) has gained popularity for cerebral perfusion imaging over contrast-enhanced methods due to its noninvasive nature, suitability for use in longitudinal studies, and concerns about links between Gadolinium-based contrast agents and nephrogenic systemic fibrosis in patients with kidney dysfunction.^3^ There is a wide range of potential applications for ASL, including acute stroke, chronic vascular disease, dementia, and assessment of tumor blood flow.

Advances in magnetic field strength, multichannel receive coils, background suppression,^4^ and the use of improved labeling schemes, such as pseudocontinuous ASL (PCASL^5^), have all improved the signal-to-noise ratio (SNR) in ASL acquisitions, priming their transition into clinical use. However, standard ASL methodologies provide information only about the total perfusion in each voxel of the brain without regard to its arterial source. An apparently normal perfusion map may be the result of efficient collateral flow (e.g., around the circle of Willis), masking the presence of disease. In addition, the cause of any observed perfusion deficit cannot be accurately assigned to a particular feeding artery due to common variations in the morphology of the cerebral vasculature.^6^ Vessel-selective information may also be of use in assessing the arterial supply to lesions such as tumors.

To address this, a number of ASL techniques capable of generating perfusion maps arising from individual arteries have been developed. These include methods that label blood in a single artery^7, 8, 9, 10, 11^ and those that label multiple arteries before separating the vascular territories in postprocessing.^12, 13, 14, 15^ The vessel-encoded PCASL (VEPCASL) approach of Wong^14^ benefits from the comparatively high SNR of the PCASL labeling scheme along with the flexibility of tagging arteries within a single plane. This is in contrast to the use of pulsed ASL techniques for vessel-selective ASL that require a complex planning procedure to position a three-dimensional (3D) slab over the artery or arteries of interest. If the vascular geometry is such that an encoding scheme can be designed which ‘tags' or ‘controls' each artery an equal number of times, then theoretically the SNR of the VEPCASL technique should match that of standard PCASL,^14^ although to our knowledge this has not yet been shown experimentally.

Thus far the VEPCASL approach has been implemented using a single postlabeling delay (PLD). This necessitates the choice of a long PLD to ensure the entire bolus has arrived at the tissue,^16^ during which the labeled blood decays considerably, reducing the SNR. It also removes the possibility of estimating the bolus arrival time (BAT) of the blood in each voxel by fitting a kinetic model^17^ to the data, which may be an interesting physiologic parameter in its own right.^18^ In addition, with separate signals from each artery it would be possible to fit the kinetic model to each component. This may produce more accurate cerebral blood flow (CBF) estimates than a standard, nonselective ASL acquisition in voxels fed by multiple arteries with different BATs, such as in patients with a significant collateral flow.

The aim of this study was to evaluate multi-PLD VEPCASL for quantitative cerebral perfusion imaging and to assess its advantages and disadvantages relative to standard PCASL, building on the work previously presented in abstract form.^19^ Simulations were performed to determine whether VEPCASL could produce more accurate CBF estimates than standard PCASL when multiple arteries contribute to perfusion in the voxel. Estimates of CBF in healthy volunteers from the two techniques were then compared to determine whether there is any systematic bias in VEPCASL relative to PCASL. The SNR of perfusion maps derived from the two techniques was also assessed and compared with theoretical expectations.

## Materials and methods

### Simulations

To test the hypothesis that VEPCASL would produce more accurate CBF estimates than standard PCASL in voxels supplied by multiple arteries, simulations of the general kinetic model for ASL^17^ were performed using Matlab (Mathworks, Natick, MA, USA). It was assumed that the simulated voxel was supplied by two arteries. Blood from the first artery had a fixed BAT, *Δt*, equal to 1 second. The BAT of blood from the second artery was varied over the range of 0.5 to 2 seconds. The total CBF was fixed at 60 mL/100 g per minute, but the ratio of CBF contributed by the two arteries was varied between 1:4 and 4:1. The simulated PCASL signal was then calculated by summing the signals from these two arterial components.

Two different SNR regimes were simulated to ascertain whether the observed trends were a result of the perfusion signal being lost within the noise floor, or whether they represented systematic errors. Here, we define SNR using perfusion images that have been averaged over all repeats and all PLDs. The ‘signal' is defined as the average gray-matter (GM) perfusion signal and the ‘noise' as the standard deviation in a background region of interest. The two regimes tested were a ‘high' SNR of 250, and a more realistic ‘low' SNR of 30, comparable to that measured in our experiments (see Results). Note that to make the simulations as realistic as possible the absolute noise level was kept constant between PCASL and VEPCASL signals. Since the PCASL signal is always larger than the VEPCASL signal, the relative noise level is higher for VEPCASL than for PCASL. Other parameters used in these simulations were set to those chosen for the experiments (described below), as listed in Table 1.

For each BAT, CBF ratio and SNR mentioned above the simulated data were generated 50 times with different random noise in each case. These simulated data were analyzed as described in ‘Analysis' section below, with the exception that no macrovascular component was considered here. The mean and standard deviation values of the resulting total CBF were then calculated.

### Experiments

Seven healthy volunteers (two female, mean age 28.9, range 25 to 37) were recruited and scanned on a 3T TIM Verio system (Siemens Healthcare, Erlangen, Germany) with a 32-channel head coil. All experiments were performed under an agreed technical development protocol approved by the Oxford University Clinical Trials and Research Governance office, in accordance with International Electrotechnical Commission and United Kingdom Health Protection Agency guidelines. A 3D multislab time-of-flight (TOF) angiography sequence was performed to enable labeling plane selection and vessel localization.

**Figure 1:**
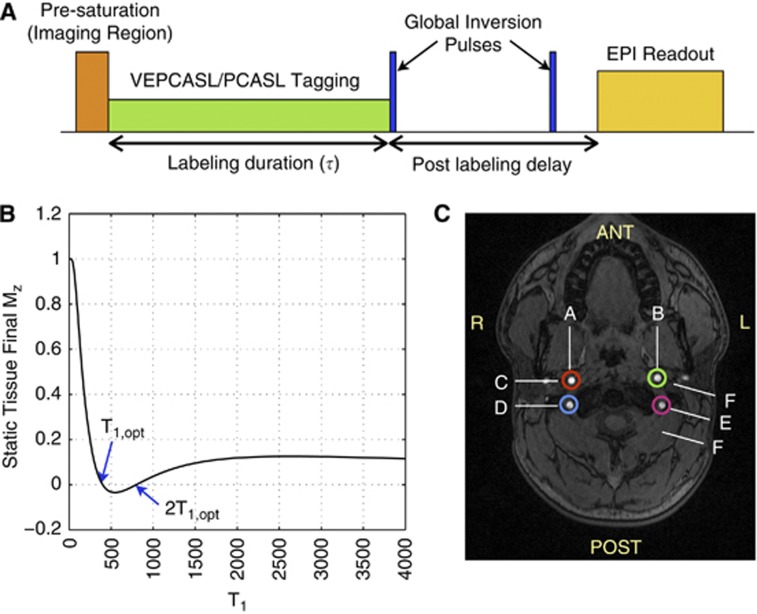
(**A**) Schematic sequence diagram. Note that the timing of the first global inversion pulse is constrained by the (vessel-encoded) pseudocontinuous arterial spin labeling ((VE)PCASL) pulse train; (**B**) Simulated longitudinal magnetization at the start of the echo planar imaging (EPI) readout for postlabeling delay (PLD)=1 second and optimum *T*_1_ (*T*_1,opt_)=390 ms. Tissues at *T*_1,opt_ and *2T*_1,opt_ are nulled perfectly but those with longer T_1_ values are also largely suppressed; (**C**) A Time-of-flight (TOF) axial slice through the neck showing a typical labeling plane. The four main brain-feeding arteries are circled in color: red=right internal carotid artery (RICA), green=left internal carotid artery (LICA), blue=right vertebral artery (RVA), and magenta=left vertebral artery (LVA). For VEPCASL, left–right encodings contrast vessels aligned with line A versus those at line B, anterior–posterior encodings contrast lines C and D and diagonal encodings contrast lines E and F. Note that F is shown twice due to the periodic nature of the encoding function.

A schematic of the ASL pulse sequence is shown in Figure 1A. Both standard PCASL and VEPCASL acquisitions were performed in each subject and shared a common labeling plane positioned ∼8 cm below the circle of Willis, through the proximal V3 segment of the vertebral arteries (VAs). In this plane, the four main brain-feeding arteries all run in the inferior–superior direction and form an approximately rectangular arrangement in the axial plane (Figure 1C). Other than the vessel-encoded preparation all parameters were kept constant between the two scans, as listed in Table 1. Pseudocontinuous ASL was achieved using 600 *μ*s duration Gaussian RF pulses once per ms over the labeling duration (*τ*) of 1.4 seconds. A single-shot echo planar imaging readout was used with repetition time (TR)=4.05 seconds and echo time (TE)=14 ms. Slices were acquired sequentially from inferior to superior, giving whole brain coverage with voxel size=3.4 × 3.4 × 5 mm. Images were acquired in separate blocks for six PLDs, ranging from 0.25 to 1.5 seconds, giving a total acquisition time of 6.5 minutes.

Background suppression was achieved by combining a water suppression enhanced through *T*_1_ effects presaturation module (similar to Golay *et al*^8^) applied to the imaging region with two global hyperbolic secant inversion pulses that followed the PCASL pulse train, which were timed^21^ to perfectly null tissues with *T*_1_ equal to *T*_1,opt_ or *2T*_1,opt_. Due to the restricted time available to play out the global inversion pulses, *T*_1,opt_ is bounded by an upper value that is PLD dependent. We therefore chose the largest *T*_1,opt_ available up to a maximum of 500 ms (see Table 1), as used by Günther *et al.*^21^ Although this gives imperfect suppression of static tissue for some PLDs, simple simulations show that tissues with *T*_1_ greater than *2T*_1,opt_ are still effectively suppressed (Figure 1B).

Vessel encoding of the right and left internal carotid arteries (ICAs) (RICA and LICA) and right and left VAs (RVA and LVA) was performed with eight paired encoding cycles (see Figure 1C): nonselective tag and control; two left–right encodings tagging first the arteries on the right while controlling those on the left, then tagging those on the left while controlling those on the right; two anterior–posterior encodings tagging first the anterior arteries while controlling the posterior arteries, then tagging the posterior arteries while controlling the anterior arteries; and finally two diagonal encodings where first the RICA and the LVA were tagged while the periodic nature of the encoding pattern allowed both the LICA and RVA to be controlled, then the LICA and RVA were tagged while the RICA and LVA were controlled.

Using the encoding matrix description of Wong^14^ this set of encoding cycles gives rise to measured data in a single voxel, **y**, such that for a perfectly rectangular arrangement of arteries within the labeling plane:


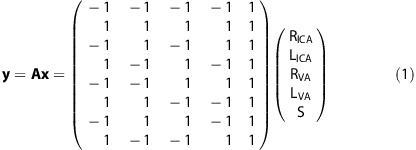


where **A** is the encoding matrix and **x** is the vector of the signals arising from the right (R) and left (L) ICAs and VAs as well as static tissue (S). This encoding matrix is full rank and has a theoretical SNR efficiency equal to that of a standard PCASL acquisition because each artery is tagged and controlled for an equal number of times.^14^ To ensure a fair comparison between VEPCASL and PCASL, the same number of volumes (96) was acquired for both techniques, meaning that a greater number of averages were acquired for PCASL at each PLD.

Additional calibration scans were acquired using identical imaging parameters to the PCASL and VEPCASL sequences, but with background suppression and labeling turned off and a longer TR (6 seconds). Three volumes after dummy scans were acquired in two separate calibration scans that used both the head coil and the body coil for signal reception. These calibration images allowed the PCASL and VEPCASL data to be corrected for the uneven spatial sensitivity profile of the head coil and facilitated calibration of CBF in absolute units (see ‘Processing' section). A *T*_1_-weighted structural image was also acquired for registration and tissue segmentation.

### Processing

**Figure 2:**
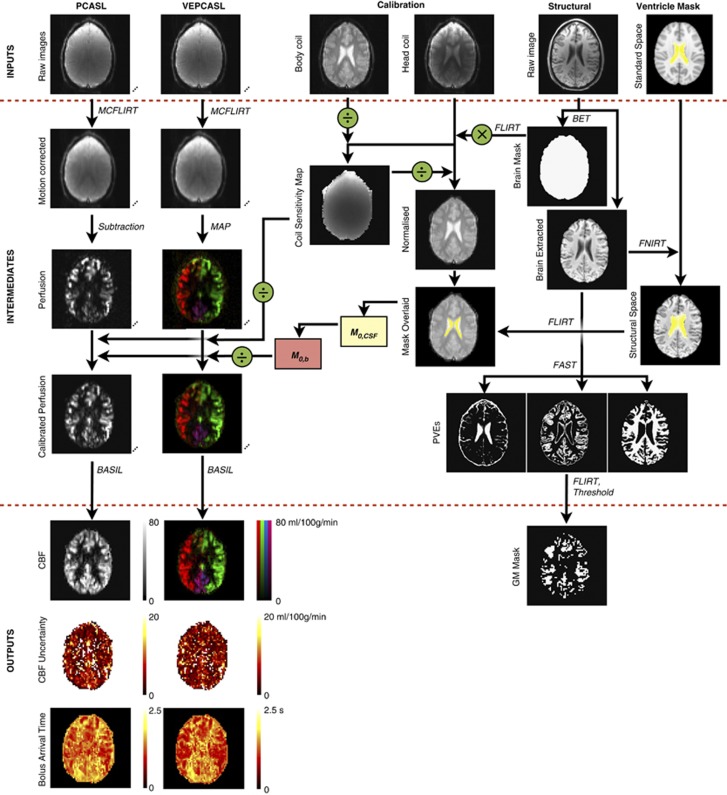
Overview of the processing performed to obtain quantitative cerebral blood flow (CBF), CBF uncertainty and bolus arrival time (BAT) maps from the vessel-encoded (VE) and standard pseudocontinuous arterial spin labeling (PCASL) data. The VEPCASL data give rise to one map per feeding artery, but these are shown together here for brevity. Partial volume estimates (PVEs) derived from the structural image were used to generate a gray matter (GM) mask used in subsequent analyses (see Results). Time-series data are represented with three dots. Various FMRIB Software Library (FSL^25^) tools were used for brain extraction (BET^26^), linear (FLIRT^22^) and non-linear (FNIRT^27^) registration, motion correction (MCFLIRT^22^), segmentation (FAST^28^) and non-linear model fitting of ASL data (BASIL^29^).

A schematic of the processing stages is shown in Figure 2. The PCASL and VEPCASL raw images were motion corrected^22^ using the head coil calibration image as a reference, before averaging across repeats. A pairwise control minus tag subtraction was then performed on the PCASL data to generate a perfusion image at each PLD. The separation of signals arising from each feeding artery in the VEPCASL data was performed using a maximum *a posteriori* (MAP) solution (method BT3)^23^ to the general Bayesian framework^24^ with two vessels per class. This approach can account for rigid subject motion between the planning TOF and VEPCASL acquisitions. For the purposes of the SNR comparison, this processing was repeated with a matrix inversion (MI) approach (see Chappell *et al*^23^), assuming no motion between the TOF and VEPCASL acquisitions.

The calibration images were time averaged before dividing the head coil image by the body coil image to give an estimate of the receive coil sensitivity map. This map was used to correct the perfusion and head coil calibration images to prevent bias in the final quantitative parameter maps.

Signal calibration was performed using cerebrospinal fluid (CSF) as a reference (similar to MacIntosh *et al*^30^). First, a standard (MNI152) space ventricle mask was nonlinearly registered^27^ to the subject's structural image, followed by linear registration^22^ to the ASL calibration image and slight erosion to prevent partial volume effects. The mean CSF signal within the ventricles was calculated and corrected for the *T*_1_ and *T*_2_* of CSF at the TR and TE used, to determine the equilibrium magnetization, *M*_0,CSF_. This value was corrected for the *T*_2_* and relative proton density of blood,^31^ the density of brain tissue (to allow CBF quantification in units of mL/100 g per minute), and the inversion efficiency of the PCASL pulse train (see Table 1), to obtain an estimate of the effective equilibrium magnetization of blood, *M*_0,b_. Note that the appropriate *T*_2_* value used to correct *M*_0,b_ depends on whether the labeled water still resides in the vascular compartment, or whether it has exchanged into tissue. In practice, these values do not yield a significantly different result at the short TE used for this experiment. The PCASL and VEPCASL perfusion images were divided through by the effective *M*_0,b_ value to complete the calibration.

A nonlinear fit to the general ASL kinetic model^17^ was performed for all voxels within a brain mask using a variational Bayes approach,^29^ accounting for the differences in acquisition time across the slices. To minimize the TE, flow crushers were not used in these experiments. As a result, the macrovascular component of the signal, which describes blood that is passing through but not contributing to perfusion in that voxel, was modeled as part of the analysis,^32^ preventing bias in the resulting CBF estimates. For the VEPCASL data, fitting was performed for each feeding artery separately. This analysis produces maps of absolute CBF and BAT and their associated uncertainty in the form of a variance value in each voxel for each parameter. To obtain a single BAT image from VEPCASL data to simplify subsequent analyses, the ‘weighted BAT' was calculated in each voxel by summing the BAT from each feeding artery weighted by the CBF fraction contributed by that artery.

Segmentation of white matter, GM, and CSF was performed^28^ using the structural image. The resulting GM partial volume estimate was transformed into the space of the ASL data before being thresholded at 0.5 to generate a GM mask for use in subsequent analyses.

### Statistics

To determine whether there was any systematic bias in the total CBF estimates derived from VEPCASL compared with those from PCASL, a linear regression was performed on each subject for all voxels within a whole brain mask. Due to the relatively high and variable uncertainty on both CBF estimates, standard linear regression tools, which minimize the sum of squared differences between two variables, are not appropriate. We therefore chose to minimize a modified version of the *χ*^2^ function, defined as:


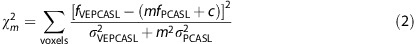


where *f* and *σ* are the total CBF estimate and its uncertainty, respectively, derived from PCASL or VEPCASL data, and *m* and *c* are the fitted slope and intercept of the line of best fit, respectively. The numerator represents the square of the difference between the observed and expected VEPCASL CBF estimate, and the denominator is the square of the uncertainty in this difference. If the two CBF estimates are equivalent, then we expect *m*≈1 and *c*≈0.

Comparison of the SNR of each technique was performed using perfusion images, considering each VEPCASL vascular territory image separately and averaging over all repeats and PLDs. The mean perfusion signal was calculated within the GM mask, and divided by the standard deviation of the noise in a background region of interest. Since each vascular territory only covers a portion of the brain, the mean signal was only calculated in those GM voxels where the feeding artery under consideration was the dominant supplier (i.e., contributed the highest mean signal of all the feeding arteries). To ensure a fair comparison, the PCASL SNR was calculated using an identical mask for each vascular territory. Finally, a separate comparison was performed in which the VEPCASL data were first summed across all feeding arteries before the SNR was calculated within all GM voxels.

## Results

**Figure 3:**
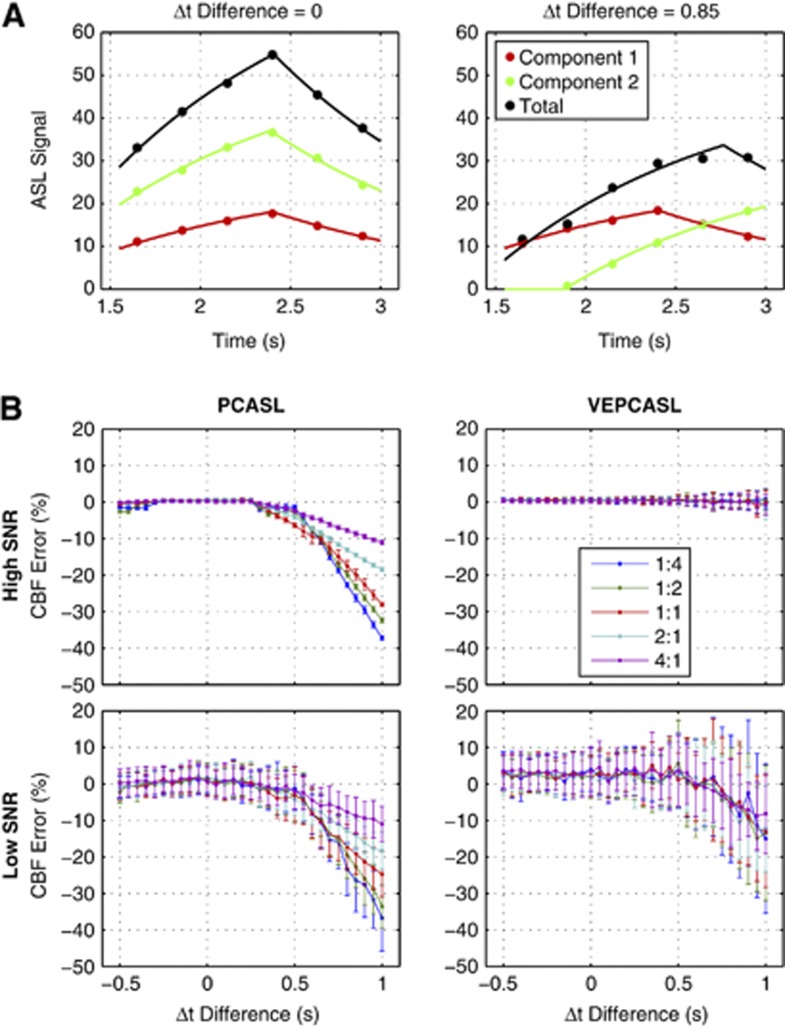
Simulation results: (**A**) Example high signal-to-noise ratio (SNR) simulated data (circles) and fits (lines) for a voxel fed by two arteries. When the arrival times of the two components are the same, both vessel-encoded (VE) and standard pseudocontinuous arterial spin labeling (PCASL) estimate the cerebral blood flow (CBF) correctly (left). When there is an arrival time difference, PCASL begins to underestimate the CBF, by 25% in this case (right); (**B**) CBF errors (mean and standard deviation) for a voxel fed by two arteries using PCASL and VEPCASL techniques. The mean and standard deviation in the CBF error are shown for a range of bolus arrival time (BAT) differences, CBF ratios (see legend) and both high and low SNR data. PCASL systematically underestimates the CBF when the BAT difference is >0.25 second. When the SNR is high, VEPCASL gives very accurate CBF estimates. When the SNR is reduced, VEPCASL begins to underestimate the CBF from the late arriving bolus, which becomes submerged in the noise floor, but the mean errors remain smaller than PCASL.

Examples of results from the simulations are shown in Figure 3A. As expected, when the BATs of the two arterial components feeding the simulated voxel are equal, the PCASL perfusion signal (total) has the same shape as the two VEPCASL signals (components 1 and 2), resulting in an accurate estimation of CBF. However, when there is a difference in the BAT between the two components, an accurate CBF estimate can no longer be extracted from the PCASL data. For VEPCASL, the two components are fitted separately, so the total CBF estimate is unaffected and remains accurate.

A summary of the simulation results is shown in Figure 3B. In the high SNR regime, PCASL systematically underestimated the CBF when the difference in arrival time between the two arterial feeders was greater than ∼0.25 second. The CBF error was greater when the delayed component contributed a larger amount to the total perfusion: errors up to 37% were found to occur over the range of parameters tested here. In contrast, VEPCASL produced accurate CBF estimates over the entire range of parameters tested here when the SNR was high. The standard deviation in the total estimated CBF increases when one arterial component is considerably delayed because this component experiences a greater degree of *T*_1_ decay, lowering the SNR. In addition, when the delay is considerable, many of the simulated data points occur before the delayed blood has arrived, reducing the average perfusion signal and therefore increasing the error in the CBF estimate.

Similar trends were seen in the more realistic ‘low' SNR regime. The systematic underestimation of CBF was the same for PCASL, although the spread of results is greater due to the higher noise level. Vessel-encoded pseudocontinuous ASL produced reasonably accurate CBF estimates for small differences in the BAT of the two arterial components, although when blood from one artery was delayed by more than ∼0.6 second, there was an underestimation of the total CBF. However, the mean errors were considerably smaller than those for PCASL and do not appear to have a strong dependence on the CBF ratio contributed by the two arterial components.

**Figure 4:**
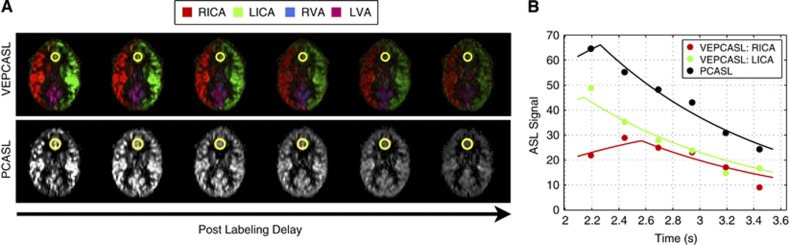
Example vessel-encoded (VE) and standard pseudocontinuous arterial spin labeling (PCASL) data: (**A**) Images of one slice in one subject at each postlabeling delay. For VEPCASL, the arterial source of the signal is representing using color, as shown in the legend; (**B**) data (circles) and fits (lines) in the encircled voxel shown in panel **A**. Only VEPCASL data from the dominant internal carotid artery (ICA) components are shown here for clarity. Blood from the right ICA (RICA) arrives later than blood from the left ICA (LICA), causing underestimation of the total cerebral blood flow (CBF) for PCASL (66.5 mL/100 g per minute) compared with VEPCASL (75.7 mL/100 g per minute).

Example perfusion images at each PLD are shown in Figure 4 for both PCASL and VEPCASL in the same subject. In this subject, the right anterior cerebral artery territory is supplied by both the RICA and the LICA. In the highlighted voxel, it can be seen that blood from the RICA is somewhat delayed relative to blood from the LICA, causing PCASL to underestimate the CBF here. However, in these healthy volunteers there were few such voxels showing significant differences in the BAT: for voxels within the brain masks only 2.0% had significant (*P*<0.01) perfusion from more than one artery and BAT differences greater than 0.5 second. This value decreases to 0.3% for BAT differences greater than 1 second.

**Figure 5:**
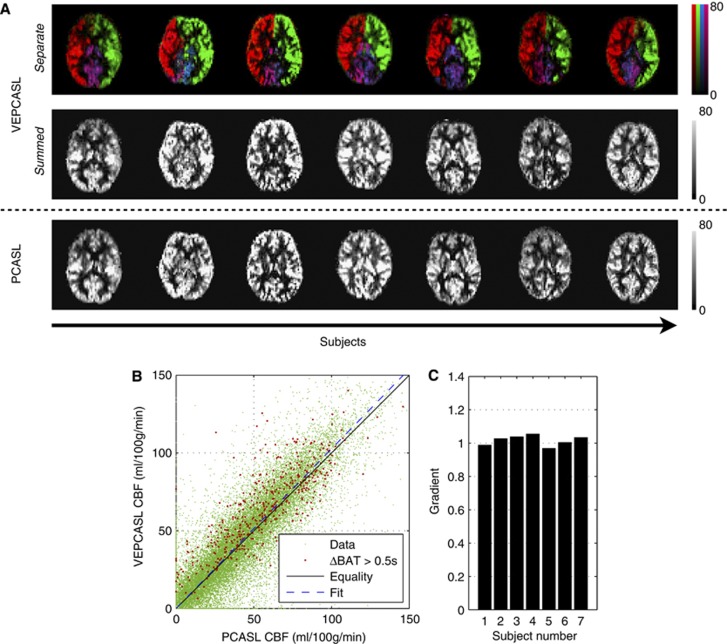
Comparison of cerebral blood flow (CBF) estimates from vessel-encoded (VE) and standard pseudocontinuous arterial spin labeling (PCASL): (**A**) Central slice of the CBF map for each subject in this study (in units of mL/100 g per minute) with VEPCASL images shown as separate components (in color) as well as summed across all feeding arteries, to aid comparison; (**B**) Correlation between the two methods for one subject for all voxels within a brain mask with the ideal equality line and line of best fit overlaid (note that PCASL tends to underestimate the CBF in voxels supplied by multiple arteries whose bolus arrival time (BAT) differences are greater than 0.5 second, plotted separately here); (**C**) Gradient of the line of best fit for each subject. Intercept values are too small to be shown here (<10^−6^ mL/100 g per minute).

Figure 5A shows example VEPCASL and PCASL CBF maps in each subject, highlighting the good image quality obtained in all cases and the qualitative similarity between the two methods. An example of the correlation analysis performed in one subject is shown in Figure 5B, showing the high degree of correlation between the CBF estimates of the two methods and a line of best fit that is very close to the ideal line of equality. In voxels fed by multiple arteries with a BAT difference of greater than 0.5 second it can be seen that there is a tendency for PCASL to underestimate the CBF, as expected from the simulations above. These voxels are therefore excluded from the correlation analysis.

The gradient of the line of best fit for each subject is plotted in Figure 5C, with mean±standard deviation (s.d.)=1.02±0.03. A Student's *t*-test confirmed that these values are not significantly different from one (*P*=0.22). In addition, the intercepts of these lines of best fit were very small (mean±s.d.=(4.2±1.8) × 10^−7^ mL/100 g per minute), showing that there is no systematic bias in the CBF estimates from VEPCASL relative to PCASL. A similar result was obtained by considering the mean GM CBF across all subjects from PCASL (53.9±7.2 mL/100 g per minute) and VEPCASL (55.0±8.8 mL/100 g per minute), where no significant difference was found (*P*=0.19).

**Figure 6:**
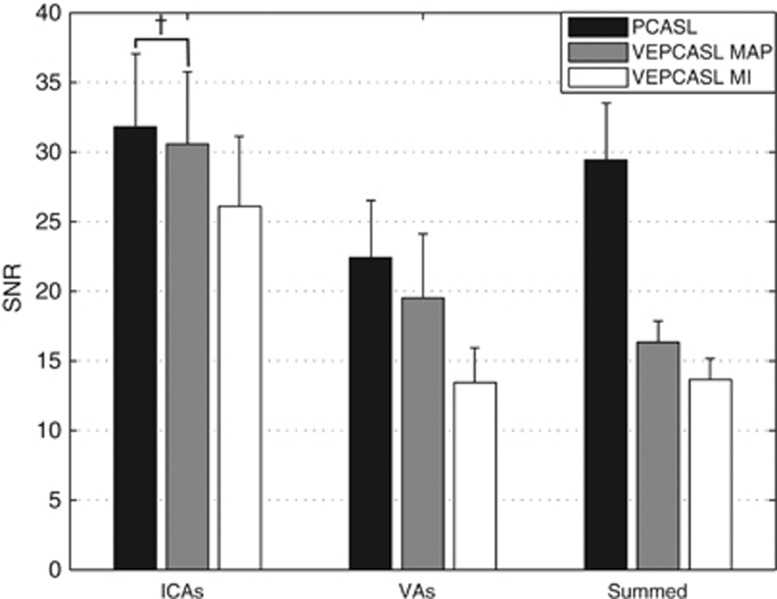
Signal-to-noise ratio (SNR) of vessel-encoded (VE) and standard pseudocontinuous arterial spin labeling (PCASL) perfusion images within a GM mask, averaged over all postlabeling delays. The mean and standard deviation values across subjects are displayed here. Within each internal carotid artery (ICA) or vertebral artery (VA) vascular territory the SNR of VEPCASL with maximum *a posteriori* (MAP) processing is close to that of PCASL. When a standard matrix inversion (MI) analysis is used, the SNR is significantly lower than that of PCASL, highlighting the improvement obtained with the Bayesian approach. However, if the perfusion signal in each voxel was first summed across all feeding arteries, then the VEPCASL SNR decreased to approximately half that of PCASL (see Discussion). All differences within each group were significant (*P*<0.05 using a paired Student's *t*-test) except those marked with a dagger (†).

A comparison of the SNR between PCASL and VEPCASL perfusion images is shown in Figure 6. When MAP processing was used, the SNR of VEPCASL was equivalent to PCASL in territories supplied mainly by one artery (ICAs), but lower in territories with mostly mixed supply (VAs). In addition, perfusion maps resulting from the MI processing had lower SNR than those obtained with MAP processing.

When the perfusion signals from all feeding arteries were summed, somewhat counterintuitively the SNR of VEPCASL decreased to about half that of PCASL. However, this effect did not appear to propagate through to the total CBF estimates: there was no significant difference in the standard deviation of the total CBF within GM, averaged across subjects, between the two methods (29.2 mL/100 g per minute for PCASL versus 29.5 mL/100 g per minute for VEPCASL, *P*=0.57).

## Discussion

In this study, we have shown that VEPCASL is a viable alternative to PCASL for CBF quantification, but with the added benefit of vessel-selective information: simulations showed that VEPCASL can produce more accurate CBF estimates in regions with mixed supply and experimentally the two methods gave identical CBF estimates with comparable SNR in the same scan time.

Figure 3 shows that considerable errors can arise in standard ASL acquisitions when a voxel is fed by multiple arteries with different BATs, as might be the case in patients with collateral flow. Vessel-encoded pseudocontinuous ASL does not have this systematic underestimation since both boluses are accurately modeled. However, in a realistic SNR regime both methods begin to underestimate the CBF when the BAT of one feeding artery is quite large and the signal begins to get lost in the noise floor. This effect is seen in both PCASL and VEPCASL simulations if the SNR is reduced further, even when the BATs are identical (data not shown).

The variability in the CBF estimates is larger for VEPCASL than for PCASL in these simulations. This is likely to be due to the relative noise being larger for VEPCASL, since the signal is split between the two arterial components but the absolute noise level is the same. The uncertainty in the total CBF, which incorporates the uncertainty from both components, is therefore increased, although the mean estimates remain more accurate. This higher relative noise also causes a slight overestimation in the total CBF for VEPCASL in the low SNR regime, even when both components have short arrival times. This is because the BAT prior is set with a mean value of 1.3 seconds. When the data are noisier, this prior has a bigger effect, causing an overestimation in the BAT and thus the CBF is slightly overestimated. However, this is a very small effect relative to measurement errors.

The perfusion images generated for this study are free from obvious artifacts in all subjects (e.g., see Figure 4A), leading to good fits to the kinetic model (Figure 4B). It is worth noting here that the use of multiple PLDs is sometimes avoided, since fewer averages at each PLD are consequently acquired for the same scan time. This leads to a reduction in the SNR of the averaged perfusion image at each PLD. However, all PLDs are used to estimate the CBF, so in essence the SNR lost in the individual perfusion images at each PLD is regained during the fitting procedure, assuming a plausible range of PLDs is adopted. The use of PCASL with a relatively long labeling duration ensures that even at short PLDs there is a significant perfusion signal, so this does not detract from the overall SNR, as might be the case with pulsed ASL methods. Finally, the use of multiple PLDs ensures that the CBF estimates are insensitive to the BAT and therefore the use of a single long PLD is not required.^16^ As a result, the average signal strength is increased, improving the overall SNR.

It was demonstrated that there is no systematic bias in the CBF estimates derived from VEPCASL relative to those from PCASL in these healthy volunteers where there is little collateral flow or delayed blood arrival. The correlation between the two methods appears to be strong (Figure 5B), with lines of best fit having gradients close to one (Figure 5C). However, in the minority of voxels supplied by multiple arteries with differing BATs, the underestimation of CBF by PCASL predicted in simulations was apparent. The small deviations from equality in other voxels could arise from minor registration errors as well as from the noisiness of the data. These fits also have very small intercepts (<10^−6^ mL/100 g per minute) which could, in part, be due to a number of voxels within the brain mask having zero, or very little, perfusion (e.g., in the ventricles or at the edges of the brain).

The SNR of VEPCASL with MAP processing was found to be equal to that of PCASL in voxels supplied by a single artery (within the ICA territories), as was expected from the ideal encoding matrix (Equation 1). In the VA territories, the SNR of VEPCASL was a little lower than that of PCASL. This might be expected, since the two VAs fuse to form the basilar artery that supplies the two posterior cerebral artery territories. Thus, many of the voxels fed by one VA have significant contributions from the other VA, decreasing the mean signal from each and therefore the SNR. Due to nonideal vascular geometry in some subjects, the actual encoding matrix deviated from the ideal case, lowering the SNR obtained with an MI approach. However, much of this is regained with the Bayesian MAP technique, which considers only a subset of the encoding matrix at a time, helping to boost the SNR.^24^

However, when the perfusion signals from each artery are summed, the SNR of VEPCASL decreases to about half that of PCASL. This is because when the VEPCASL images are summed, the total ‘true' perfusion signal is also summed, thereby giving the same total signal as the PCASL data. However, the noise present in each VEPCASL perfusion image is approximately equal to that in the PCASL perfusion image. Thus, for four feeding arteries the noise variance of the summed image is four times that of the individual VEPCASL perfusion images, and thus the SNR is halved. Therefore, a simple summation of the raw VEPCASL perfusion signals is detrimental to image quality.

Interestingly, this effect does not appear to propagate through to the total CBF estimates in the same way. The standard deviation of the total CBF in GM was not significantly different for VEPCASL and PCASL. Clearly, this measure encompasses CBF variations due to partial volume effects and natural variability. However, if there was a considerable difference in the uncertainty of the total CBF, then it would be expected that the standard deviation would be higher for VEPCASL. The similarity in the standard deviation of both techniques may be attributable to the kinetic model fitting procedure. Arterial components that do not contribute to perfusion are effectively suppressed by the zero-mean CBF prior. Therefore, since most voxels are only supplied by one artery, the uncertainty in the total CBF is approximately equal to the uncertainty in the dominant arterial component, which is comparable to that of PCASL.

Given these results, it is expected that the VEPCASL technique would be particularly useful for CBF quantification in patients with stenosis or occlusion of the proximal brain-feeding arteries. The ability of VEPCASL to separate out the signals arising from each feeding artery should allow more accurate CBF quantification in regions of mixed supply, which may arise due to collateral flow around the circle of Willis. In addition, the information obtained about the degree of collateral flow may be clinically beneficial for balancing the risks and benefits of potential interventions.

However, it should be noted that in patient groups the vascular geometry may be less ideal and there may be considerable motion between the planning TOF and VEPCASL acquisition. The Bayesian analysis method used here is capable of accounting for this type of motion, but the SNR of the VEPCASL technique might be reduced relative to PCASL as the achieved encoding matrix deviates further from the ideal case. Further work is required to show the suitability of this technique in patient populations.

This study had a number of limitations, however. The pulse sequence used did not include flow crushing gradients and as a result, the perfusion images contained macrovascular signal. It has previously been shown that even if flow crushers are used, there may still be some residual macrovascular signal that can bias the resulting CBF estimates.^32^ In this study, the macrovascular signal was removed at the kinetic model fitting stage of the analysis pipeline. Furthermore, through not using flow crushing gradients a shorter TE could be used than would otherwise be possible, thereby improving the SNR.

The background suppression scheme used here was suboptimal due to the restricted timing available to play out the global inversion pulses. This could be improved by incorporating the interleaved scheme of Robson *et al.*^33^ However, some residual longitudinal magnetization is required for magnitude subtraction to be performed, and also for better conditioning of the motion correction algorithm. In addition, this background suppression scheme is only optimized for the first acquired slice. A 3D readout such as 3D-Gradient and Spin Echo (3D-GRASE)^21^ would therefore be preferable, although the through-slice blurring of this technique is not ideal for vascular territory imaging.^34^

It should be noted that tissues with *T*_1_ between *T*_1,opt_ and 2*T*_1,opt_ will be inverted at the start of the readout (see Figure 1B). This could cause problems when analyzing magnitude images since inverted blood flowing in will add to, rather than subtract from, the magnitude of the total measured signal, leading to a negative perfusion signal. For the maximum value of *T*_1,opt_ used here (500 ms) white matter, with *T*_1_ approximately equal to 1 second at 3 T, will be close to this problematic region for some PLDs. However, this value of *T*_1,opt_ has previously been used successfully for ASL experiments,^21^ probably because we are primarily interested in the perfusion of GM, which has a longer *T*_1_. In addition, since *T*_1_ recovery continues during the readout, inverted tissue magnetization will become positive in later slices. Simulations similar to those in Figure 1B reveal that for the timings used in this study inverted tissue magnetization is only present within the first slice, with all other slices containing only positive tissue magnetization.

In the simulations, it was assumed that the PCASL data were always fitted using a single vascular component model. Fitting two components could have resulted in improved CBF estimates.^35^ However, as can be seen in Figures 3A and 4B, the summed signal from two components is difficult to distinguish from a single component, particularly in the presence of noise. In addition, the number of arteries contributing to the perfusion in a given voxel would not be known *a priori*, so the practical implementation would be difficult.

Although it has been shown that VEPCASL can produce more accurate estimates of CBF in regions of mixed supply, this is only the case when each arterial source has been separately encoded at the labeling plane. For the encoding scheme used in this study, this will allow more accurate CBF quantification in the presence of primary collateral flow around the circle of Willis. However, in voxels supplied by multiple arterial branches distal to the circle of Willis (e.g., two branches of the right middle cerebral artery), the different boluses would not be distinguished.

Finally, the fitting procedure used the simplest version of the general kinetic model.^17^ The incorporation of dispersion information, either using estimates from angiographic data,^36, 37^ or by fitting to the data directly^38, 39^ might help improve the accuracy of CBF quantification. Additionally, more complex kinetic models could be used that account explicitly for microvascular and tissue compartments and the finite permeability of the capillary bed.^40, 41^

To conclude, we have shown in this study that VEPCASL produces the same CBF estimates as standard PCASL with comparable SNR, but with the addition of vessel-selective information. Simulations showed that this would improve CBF estimates in regions supplied by multiple arteries, such as those partially fed by collateral flow. The only significant disadvantages of this method are increased complexity in the acquisition set-up and image processing, along with the potential for increased sensitivity to subject motion.

## Figures and Tables

**Table 1:** Parameters used for simulations, experiments, and analysis

*Parameter*	*Value*
*Experimental*	
Radiofrequency (RF) labeling pulses	Gaussian, 600 *μ*s duration
RF labeling pulse separation	1 ms
Mean tagging gradient	0.8 mT/m
Tagging gradient during RF pulses	6 mT/m
Labeling duration (*τ*)	1.4 seconds
Postlabeling delays (PLDs)	0.25, 0.5, 0.75, 1.0, 1.25, 1.5 seconds
Background suppression *T*_1,opt_	80, 170, 280, 390, 500, 500 ms^a^
Voxel size	3.4 × 3.4 × 5 mm
Matrix size	64 × 64
Partial Fourier factor	6/8
Echo time (TE)	14 ms
Number of slices	24
Time per slice	45.2 ms
Volume repetition time (TR)	4.05 seconds
Number of volumes	96
Acquisition time	6.5 minutes
*Analysis*	
Inversion efficiency	0.88^b^
Longitudinal relaxation time of tissue (*T*_1_)	1.3 seconds
Longitudinal relaxation time of blood (*T*_1b_)	1.6 seconds
Blood/tissue water partition coefficient (*λ*)	0.9 mL/g
Longitudinal relaxation time of CSF (*T*_1CSF_)	4.3 seconds
Transverse relaxation time of CSF (*T*_2CSF_*)	750 ms
Transverse relaxation time of blood (*T*_2b_*)	50 ms
Cerebral blood flow prior (mean±s.d.)	0±10^6^ mL/100 g per minute
Bolus arrival time prior (mean±s.d.)	1.3±0.5 seconds

CSF, cerebrospinal fluid; s.d., standard deviation.

a The chosen *T*_1,opt_ value is dependent on the PLD.

b Inversion efficiency determined via simulations of the Bloch equations (as per Okell *et al*^20^), assuming laminar flow with average velocity 30 cm/s.

## Notes

MAC and TWO are authors of a pending US patent licensed to Siemens Healthcare (Erlangen, Germany) relating to the MAP processing technique used in this study.
